# Colonisation dynamics of *Listeria monocytogenes* strains isolated from food production environments

**DOI:** 10.1038/s41598-021-91503-w

**Published:** 2021-06-09

**Authors:** Jessica Gray, P. Scott Chandry, Mandeep Kaur, Chawalit Kocharunchitt, Séamus Fanning, John P. Bowman, Edward M. Fox

**Affiliations:** 1 CSIRO Agriculture and Food, Werribee, VIC Australia; 2 Food Safety Centre, Tasmanian Institute of Agriculture, School of Land and Food, University of Tasmania, Hobart, TAS Australia; 3 Biosciences and Food Technology, School of Science, RMIT University, Melbourne, VIC Australia; 4 UCD-Centre for Food Safety, School of Public Health, Physiotherapy and Sports Science, University College Dublin, Dublin, D04 N2E5 Ireland; 5 Institute for Global Food Security, Queen’s University Belfast, Chlorine Gardens, Belfast, BT5 6AG UK; 6 Department of Applied Sciences, Northumbria University, Newcastle upon Tyne, NE1 8ST UK

**Keywords:** Biofilms, Pathogens

## Abstract

*Listeria monocytogenes* is a ubiquitous bacterium capable of colonising and persisting within food production environments (FPEs) for many years, even decades. This ability to colonise, survive and persist within the FPEs can result in food product cross-contamination, including vulnerable products such as ready to eat food items. Various environmental and genetic elements are purported to be involved, with the ability to form biofilms being an important factor. In this study we examined various mechanisms which can influence colonisation in FPEs. The ability of isolates (n = 52) to attach and grow in biofilm was assessed, distinguishing slower biofilm formers from isolates forming biofilm more rapidly. These isolates were further assessed to determine if growth rate, exopolymeric substance production and/or the *agr* signalling propeptide influenced these dynamics and could promote persistence in conditions reflective of FPE. Despite no strong association with the above factors to a rapid colonisation phenotype, the global transcriptome suggested transport, energy production and metabolism genes were widely upregulated during the initial colonisation stages under nutrient limited conditions. However, the upregulation of the metabolism systems varied between isolates supporting the idea that *L. monocytogenes* ability to colonise the FPEs is strain-specific.

## Introduction

*Listeria monocytogenes* is a Gram-positive foodborne pathogen which can cause the life-threatening disease listeriosis, particularly in at-risk populations. While listeriosis is an uncommon food borne illness, in the at-risk population group covering immunocompromised, elderly, pregnant women and neonates, the mortality rate can reach as high as 30%^1–3^. As the food supply chain has become progressively more global, increased reports of multistate and international food recalls and outbreaks are occurring, therefore the need to understand *L. monocytogenes* ability to colonise and persist in food processing environments (FPEs) is paramount^4^. Traditionally the presence of *L. monocytogenes* in food products has been associated with foods like ready to eat meats, seafood products, unpasteurised milk and dairy products, however new food items like melons, various fresh, pre-cut and frozen fruit and vegetables, leafy greens, sandwiches and wraps are now being linked to *L. monocytogenes*^4^. The ubiquitous nature of this foodborne bacterium makes it difficult to control and manage, and due to this can be repeatedly introduced into FPEs^5^ and therefore efforts should be targeted towards this environment. It is not uncommon for reports of persistent strains to arise with studies describing the isolation of some strains over numerous years^6–9^. The presence of persistent strains in the FPE can act as a repetitive source of contamination and imply the cleaning and sanitation program is not always effective in their control. Persistence within the FPE is suspected to be linked to a variety of factors including resistance and tolerance to disinfectants, acid and heat applications, favourable niches due to poor facility design and condition, along with the ability to attach to a variety of surfaces and the formation of biofilms^10–13^.

Biofilms consist of microbial cells, generally multi-species, attached to each other or a surface, and surrounded by an extracellular polymeric substance which provides increased fitness to all cells within the biofilm^14–16^. Biofilms provide increased protection from cleaning agents, disinfectants and desiccation, enhances the transfer of nutrients and removal of toxic metabolites, and increases the opportunity to acquire new genetic traits^14–16^. The process of attachment and biofilm formation in *L. monocytogenes* has been reported^17–19^, however there is less consensus on what specific genes are responsible for *L. monocytogenes* ability to colonise and survive in FPEs, and it is likely a synergy of multiple mechanisms are involved. The aims of this study were to develop a simplified model system to reflect the FPE in terms of contact surface, temperature and limited nutrient availability, key conditions in FPEs. This simplified model system was then used to determine: (i) if there were any differences in the early stages of biofilm formation between *L. monocytogenes* strains isolated from various food and environmental sources for multi-locus sequence types (MLST) commonly associated with FPEs; (ii) if there are genes or phenotypes associated with the biofilm phenotype; (iii) if there are differences in expression levels of the signalling associated *agrD* gene, known to be involved in adherence, between fast and slow biofilm formers and; (iv) if there are differences in transcription levels of genes between two MLST STs both present in the slow and fast biofilm groups.

## Results

### Isolate characteristics

The strains included in this study were isolated from food or related environments, including meat, dairy, vegetable, and mixed sources, across a span of 18 years from 1998 to 2016 (Supplementary Table 1). The draft genome sizes of the 52 *L. monocytogenes* isolates ranged from 2.61 to 3.08 Mb, with the GC percentage between 37.7 and 38.1%.

### Biofilm formation on SS coupons

**Figure 1:**
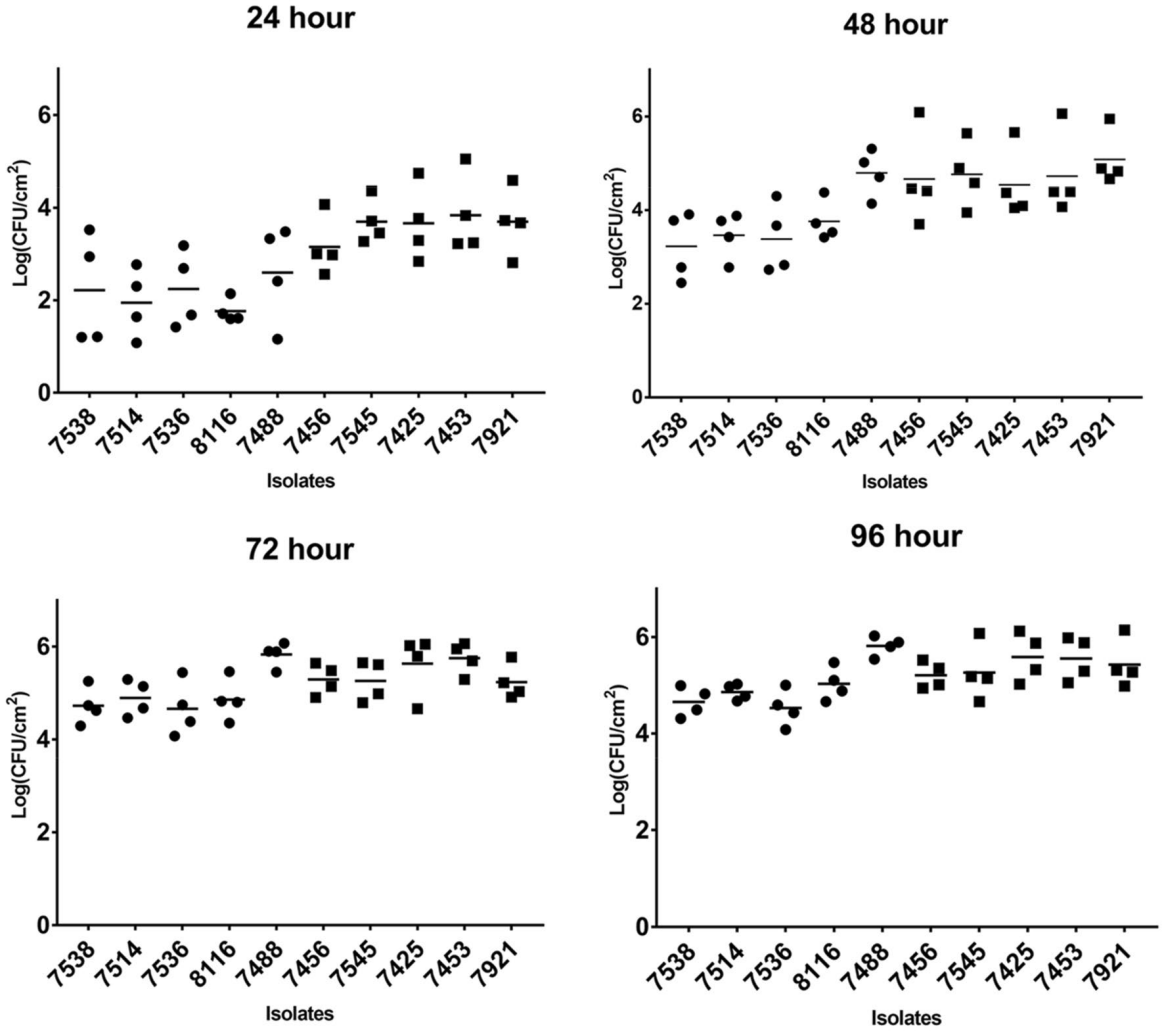
Comparison of the B10 isolates identified as displaying faster or slower biofilm formation over 96 h. Biofilm density (log_10_ CFU/cm^2^) was determined every 24 h by standard plate count. Data points represent the average of 4 biological experiment replicates. Dots, slow isolates; squares, fast isolates.

Biofilm formation was assessed on SS coupons at 24, 48, 72 and 96 h. Isolates were examined based on mean biofilm cell density (log_10_ CFU/cm^2^) with a broad distribution observed at each timepoint (Table 1) indicating all isolates were able to form biofilms. Greatest differences in mean biofilm density between strains was observed at 24 and 48 h (Supplementary Fig. 1). A fast biofilm forming group with cell densities of 3.5–4.2 log_10_ CFU/cm^2^ formed after 24 h was well separated from a slow biofilm forming isolates which only reached 1.2–1.8 log_10_ CFU/cm^2^ after 24 h (Fig. 1). This separation was less evident by 72 and 96 h with the mean cell densities being within 1.5 log_10_ when comparing isolates. The five isolates with the highest biofilm cell densities at 24 h were deemed to be the fast biofilm forming group (appended with ^F^ for clarity) consisted of 7921^F^, 7453^F^, 7425^F^, 7545^F^ and 7546^F^. The five isolates with the lowest biofilm cell densities at 24 h were considered slow biofilm formers (appended with ^S^) and included 7488^S^, 8116^S^, 7536^S^, 7514^S^ and 7538^S^; together, these fast and slow biofilm groupings make up the B10 isolates. At 24 h, three of the five isolates in the slow biofilm forming group were from Lineage I and four of the five isolates from the fast biofilm forming group were from Lineage II. Two of the isolates from the fast biofilm forming group were from MLST ST155. MLST ST101 and ST2 had an isolate in both groups whereas all the other isolates in the two groups were from different STs.

**Table 1:** Mean biofilm density (log_10_ CFU/cm^2^) range of all 52 *L. monocytogenes* biofilm isolates at sampled timepoints.

Time point (h)	log_10_ CFU/cm^2^	Variation in cell numbers (log_10_ CFU/cm^2^)
24	1.20–4.16	2.95
48	2.61–5.39	2.78
72	4.51–5.83	1.32
96	4.40–5.82	1.41

### EPS production

The ability to produce exopolymeric substances was assessed in the B10 group to investigate if these features influence the ability of isolates to attach and form biofilms faster. In this study, isolates which showed a pink phenotype at 14 °C regardless of the growth media were 7425^F^, 7453^F^, 7488^S^, 7514^S^, 7536^S^ and 7538^S^ illustrating an intermediate ability to bind Congo red and thus produce some form of EPS (Supplementary Fig. 2). A translucent phenotype was displayed by isolates 7456^F^, 7545^F^, 7921^F^ and 8116^S^ indicating they were unable to bind the Congo red dye and therefore did not produce EPS. At 37 °C most isolates displayed the same phenotype as they did at 14 °C although some changed phenotype, isolates 7453^F^ and 7488^S^ which became translucent, and 7456^F^ which produced a pink phenotype. Isolates 7536^S^ and 7538^S^ changed from the pink phenotype to translucent at 37 °C when grown in MHB. The above phenotypes were not associated with a slow or fast biofilm formation group.

### Growth rate and doubling time of B10

**Figure 2:**
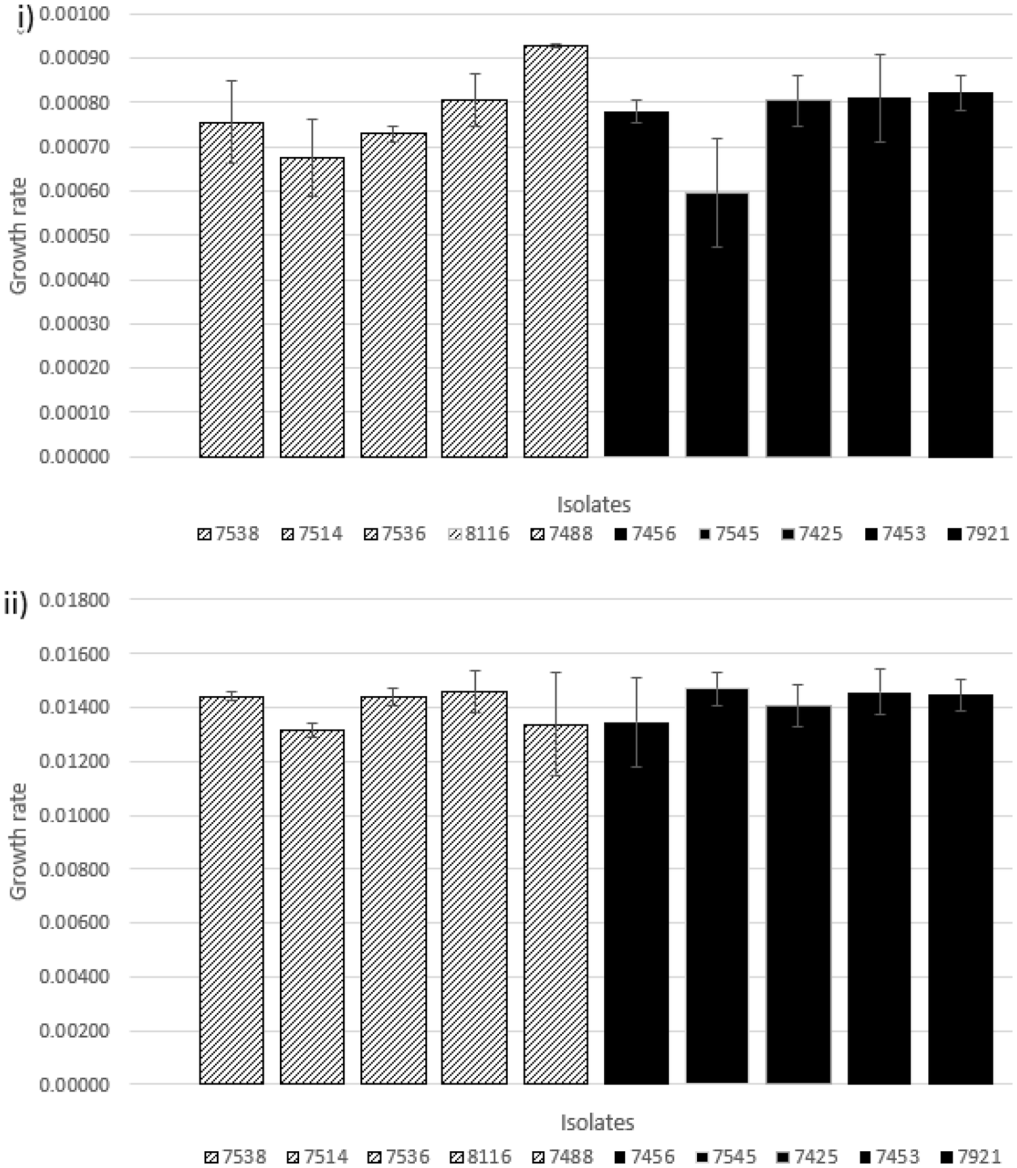
Mean specific growth rate of the B10 isolates and standard deviation at: (**i**) 14 °C in dBHI (three biological replicates) and; (**ii**) 37 °C in full BHI (two biological and seven technical replicates). Patterned bars—slow isolates; solid black bars—fast isolates.

The growth rate of the B10 isolates at 14 °C in dBHI and at 37 °C in BHI was determined (Fig. 2). At 14 °C the isolates growth rate ranged from 0.00060 and 0.00093 min^−1^. The slowest growth rate was associated with isolate 7545^F^ with the fastest growth rate belonging to isolate 7488^S^. The doubling time was also measured with a broad range of times observed (12.4–19.9 h). At 37 °C the growth rate and doubling times ranged from 0.01315 to 0.01468 min^−1^ and 43.55 to 48.61 min, respectively, reflective of typical *L. monocytogenes* growth under optimal conditions. Importantly, growth rate and doubling times were not correlated to biofilm forming ability at either temperature.

### Genome wide association study

A microbial genome wide association study was performed across the 52 isolates utilising the biofilm phenotypic data to assess if there were any genetic differences associated with biofilm formation and attachment ability. No significant single nucleotide polymorphisms (SNPs) were associated with a faster or slower biofilm formation phenotype were identified amongst the 52 isolates; similarly, no genes showed statistically significant phenotypic association. Phylogenetic association was determined by treeWAS based upon 28,414 core SNPs resulting in isolates grouping by clonal complex (Supplementary Fig. 3).

### *agrD* gene expression

**Figure 3:**
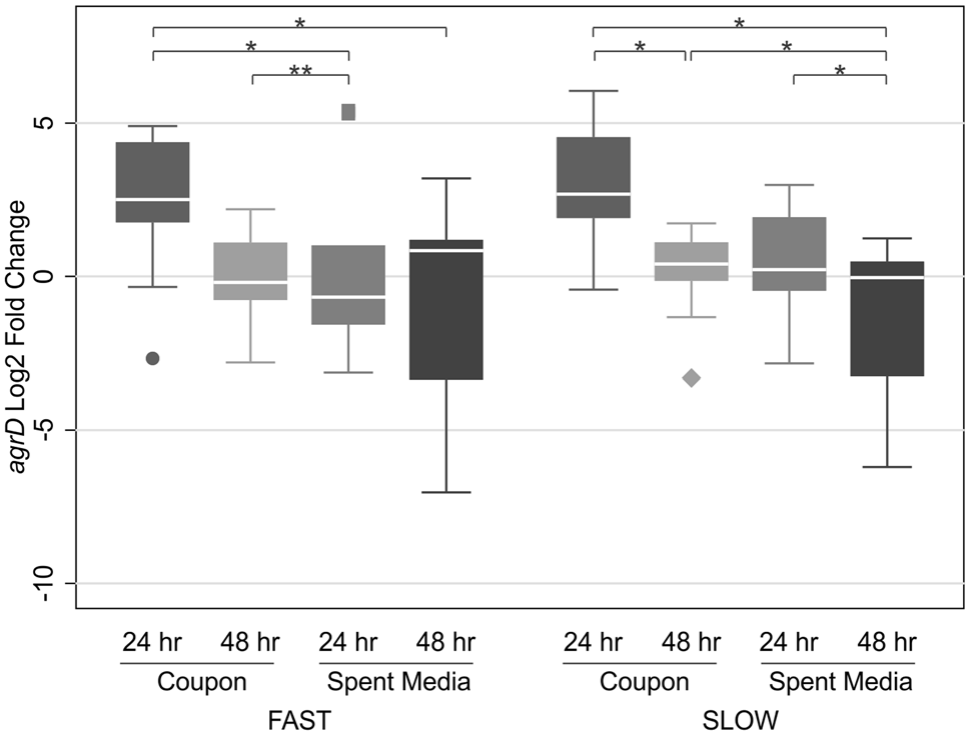
*agrD* expression (log_2_ fold change) at 24 and 48 h in coupons and spent media. No statistically significant difference in *agrD* expression between slow and fast isolates. Comparison of paired conditions or timepoints displayed significant difference, specifically C24hr Fast and SM24hr Fast (Z = 2.073, p = 0.0382), C48hr Slow and SM48hr Slow (Z = 1.992, p = 0.0464), C24hr Slow and C48hr Slow (Z = 2.490, p = 0.0128), SM24hr Slow and SM48 Slow (Z = 2.192, p = 0.0284), C24hr Fast and SM48hr Fast (Z = 2.341, p = 0.0192), C48hr Fast and SM24hr Fast (Z = 2.970, p = 0.0030) and C24hr Slow and SM48hr Slow (Z = 2.521, p = 0.0117). *p < 0.05; **p < 0.01. *C* coupon, *SM* spent media, *Z* z score, shading refers to the different experimental conditions.

The expression levels of the propeptide *agrD* was assessed using qRTi-PCR in the B10 isolates on coupons and in SM at 24 and 48 h. The Wilcoxon rank sum test indicated there was no statistically significant difference in *agrD* expression when comparing the fast and slow isolates against the independent growth conditions and timepoints. When the *agrD* expression is assessed by either paired condition or timepoint some differences are determined (Fig. 3). Notably, *agrD* expression was upregulated in the early stages of attachment and biofilm development, relative to other conditions tested.

### Transcriptional analysis

#### Differentially expressed genes (DEGs) under food production environment biofilm formation conditions

The global transcriptomic changes in biofilm formation at 24 h and 48 h in dBHI was assessed against four individual isolates with two isolates from both the slow and fast biofilm formation groups, respectively. The isolates chosen represented ST101 and ST2 with a fast and slow isolate in each ST. The number of reads ranged from 21,954,948 to 65,818,623 and were mapped to each isolate’s individual genome. A total of 494 differentially expressed genes (DEGs) were identified using a false discovery rate (FDR) of < 0.01 and log fold change (logFC) of ≥ 2 across all comparisons. Isolate 7538^S^ and ST2 at both timepoints had no DEGs which met the FDR and log_2_FC cut-off. At 24 h isolates 7453^F^, 7545^F^ and 8116^S^ had 286, 76 and 7 DEGs respectively resulting in a total of 369 up regulated DEGs. At 48 h isolates 7453^F^ and 7545^F^ had 85 and 23 DEGs respectively totalling 108 DEGs. Between ST101 there was 11 up regulated and 6 down regulated DEGs at 24 h and 48 h respectively. The DEGs were annotated in Eggnog.

#### Functional annotation of transcriptome

The clusters of orthologous groups (COGs) were used to identify the functional categories of the DEGs. The DEGs were allocated to 20 of the COG categories (Supplementary Table S2) with 19 DEGs assigned to multiple (> 1) COG categories and were therefore treated as belonging to both. Approximately a third (30%) of the DEGs were allocated to the ‘S’ COG categories ‘Function unknown’. Amongst the 24 h up regulated DEGs with functional assignments the next three prevalent COG categories are ‘G: Carbohydrate transport and metabolism’, ‘J: Translation, ribosomal structure and biogenesis’, and ‘M: Cell wall/membrane/envelope biogenesis.’ The top three amongst 48 h up regulated DEGs also includes categories ‘G’ and ‘J’ along with ‘K: Transcription’.

#### Pathways identified

**Figure 4:**
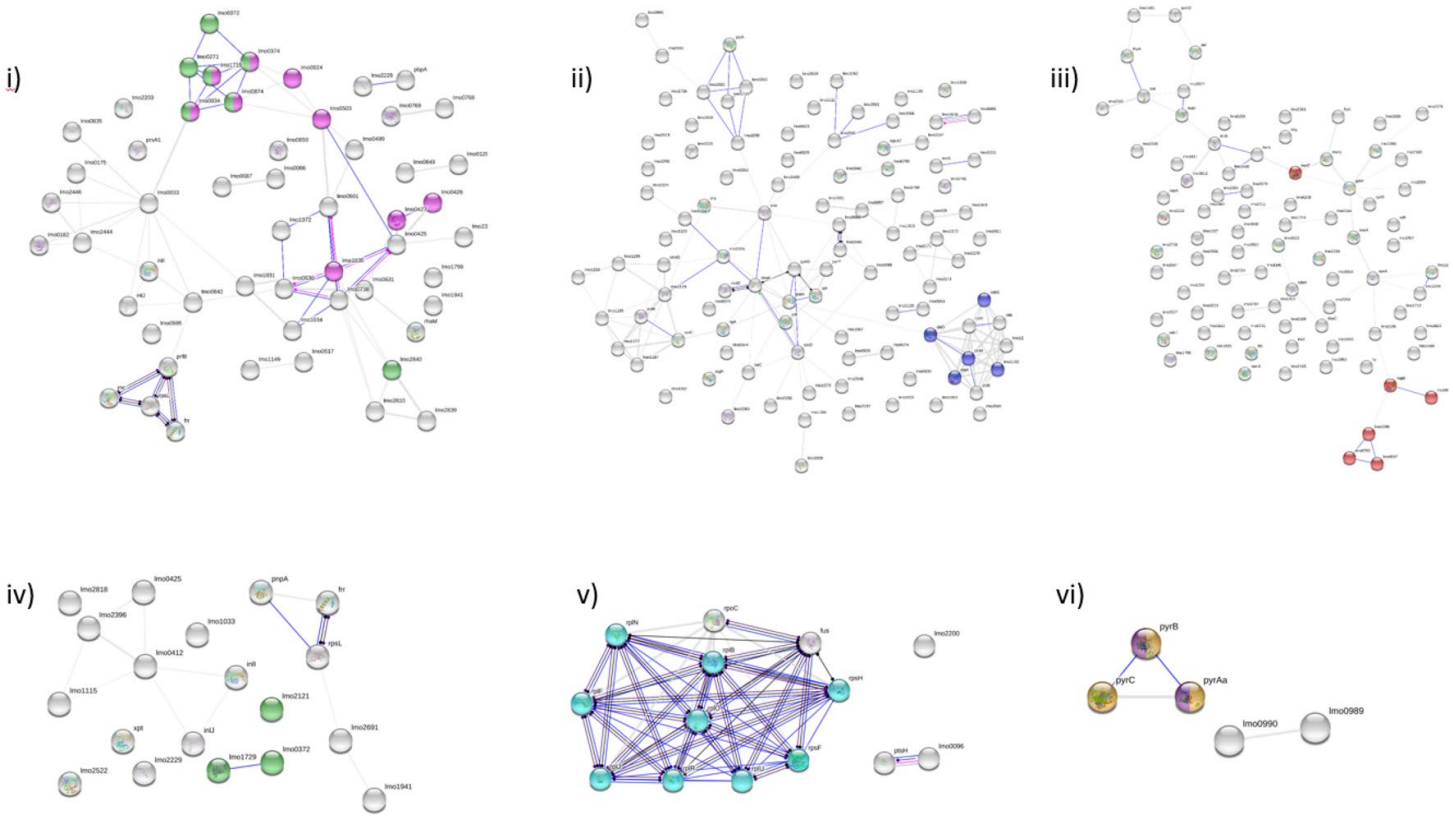
Overexpressed protein pathways in the transcriptome at 24 and 48 h in isolates 7453, 7545 and ST101 48 h. (**i** and **ii**) 7453 24 h; (**iii**) 7453 48 h; (**iv**) 7545 24 h; (**v**) 7545 48 h; (**vi**) ST101 48 h. Coloured nodes relate to overexpressed pathways: pink, phosphotransferase system; light green, starch and sucrose metabolism; dark blue, cobalamin biosynthesis; red, amino sugar and nucleotide sugar metabolism; light blue, ribosome; yellow, pyrimidine metabolism; and purple, alanine, aspartate and glutamate metabolism. Coloured lines connecting nodes relate to action type: blue, binding; black, reaction; purple, catalysis; and pink, post-translation modification. Locus tags and genes names are based upon matches to proteins in the reference genome, *L. monocytogenes* EGD-e.

The STRING database was used to identify overexpressed pathways and the molecular mode of action present within the DEGs of isolates 7453^F^, 7545^F^ and ST101 at 24 and 48 h. (Fig. 4 and Table 2). In isolate 7453^F^, the phosphotransferase system (PTS) (FC range 3.48–6.10) and starch and sucrose metabolism pathways (FC range 3.66–6.10) were overexpressed at 24 h along with cobalamin biosynthesis (FC range 3.35–5.05). The pathways for amino sugar and nucleotide sugar metabolism (FC range 2.99–3.89) were overexpressed at 48 h. The overexpressed pathway identified in isolate 7545^F^ at 24 h included starch and sucrose metabolism (FC range 2.87–3.69). At 48 h the pathway overexpressed was ribosome (FC range 3.36–4.56) associated with various RNA proteins and ribosomal domains identified. Pyrimidine metabolism and alanine, aspartate and glutamate metabolism (FC range 4.20–4.37) were pathways overexpressed for the ST101 48 h down regulated DEGs (Table 3). The upregulated DEGs of ST101 at 24 h predominately consisted of prophage genes (FC range 9.61–12.10). In addition, isolate 7453^F^ at 24 h also contain prophage up regulated DEGs (FC range 3.34–6.20). Most of the molecular action consisted of post translational modification, reaction, binding and catalysis.

**Table 2:** Overexpressed pathways in differentially* expressed genes at 24 and 48 h in *L. monocytogenes* isolates 7453^F^ and 7545^F^.

Locus tag (EGD-e)	Locus tag (this study)	Fold change	Gene	COG cat	Description	KEGG enzyme	Isolate and TP^#^
**PTS system**							
lmo1035	fig|1639.4014.peg.1355	4.22		G	PEP-dependent sugar PTS, EIIA 1		7453 24 h
lmo1719	fig|1639.4014.peg.1473	4.36		G	PTS system cellobiose-specific IIA component	2.7.1.205	
lmo0427	fig|1639.4014.peg.2115	4.77		G	PTS system, Lactose/Cellobiose specific IIB subunit		
lmo0426	fig|1639.4014.peg.2116	4.02		G	PEP-dependent sugar PTS, EIIA 2		
lmo0024	fig|1639.4014.peg.434	4.99		G	PTS system mannose/fructose/sorbose family IID component		
lmo0034	fig|1639.4014.peg.444	5.61		G	PTS system cellobiose-specific IIC component		
lmo0374	fig|1639.4014.peg.610	3.66		G	PTS system cellobiose-specific IIB component	2.7.1.205	
lmo0874	fig|1639.4014.peg.773	6.1		G	PTS system, Lactose/Cellobiose specific IIA subunit; PTS system beta-glucoside-specific IIA component		
lmo0503	fig|1639.4014.peg.936	3.48		G	PTS system galactitol-specific IIA component	2.7.1.200	
**Starch and sucrose metabolism**							
lmo0271	fig|1639.4014.peg.1428	4.47		G	Glycosyl hydrolase 1 family; 6-phospho-beta-glucosidase	3.2.1.86	7453 24 h
lmo1719	fig|1639.4014.peg.1473	4.36		G	PTS lichenan-specific enzyme IIA component; PTS system beta-glucoside-specific IIA component; PTS system cellobiose-specific IIA component	2.7.1.205	
lmo2840	fig|1639.4014.peg.393	4.82	YcjM	G	Sucrose glucosyltransferase/sucrose phosphorylase (ycjM)	2.4.1.7	
lmo0034	fig|1639.4014.peg.444	5.61		G	PTS system cellobiose-specific IIC component		
lmo0372	fig|1639.4014.peg.608	4.16		G	Glycosyl hydrolase 1 family; 6-phospho-beta-glucosidase	3.2.1.86	
lmo0374	fig|1639.4014.peg.610	3.66		G	PTS system cellobiose-specific IIB component	2.7.1.205	
lmo0874	fig|1639.4014.peg.773	6.1		G	PTS system, Lactose/Cellobiose specific IIA subunit; PTS system beta-glucoside-specific IIA component		
lmo1729	fig|1639.4024.peg.1538	3.27		G	Glycosyl hydrolase 3 family; beta-glucosidase	3.2.1.21	7545 24 h
lmo0372	fig|1639.4024.peg.670	3.69		G	Glycosyl hydrolase 1 family; 6-phospho-beta-glucosidase	3.2.1.86	
lmo2121	fig|1639.4024.peg.985	2.87		G	Trehalose and maltose hydrolases; Maltose phosphorylase	2.4.1.8	
**Cobalamin biosynthesis**							
lmo1148	fig|1639.4014.peg.281	5.05	cobS	H	Cobalamin synthase	2.7.8.26	7453 24 h
lmo1192	fig|1639.4014.peg.237	3.51	cobD	H	Adenosylcobinamide-phosphate synthase	6.3.1.10	
lmo1194	fig|1639.4014.peg.235	3.95	cbiD	H	Cobalt-precorrin-5B (C1)-methyltransferase	2.1.1.195	
lmo1191	fig|1639.4014.peg.238	3.35	cbiA	H	Cobyrinic acid c-diamide synthetase	6.3.5.11	
lmo1204	fig|1639.4014.peg.225	4.61	cbiM	P	Cobalt ECF transporter substrate-binding protein CbiM		
**Prophage related genes**							
	fig|1639.4014.peg.1757	3.84		K	BRO family, N-terminal domain; Antirepressor [Bacteriophage A118]		7453 24 h
	fig|1639.4014.peg.1796	3.38		N	Bacterial Ig-like domain 2; Protein gp13 [Bacteriophage A118]		
	fig|1639.4014.peg.2230	3.41		S	Phosphoadenosine phosphosulfate; Co-activator of prophage gene expression IbrA		
	fig|1639.4014.peg.2484	5.39		S	Phage protein		
	fig|1639.4014.peg.1804	4.2		S	Putative short tail fibre [Bacteriophage A118]		
	fig|1639.4014.peg.1780	4.16		S	Protein of unknown function (DUF2481) [Bacteriophage A118]		
	fig|1639.4014.peg.2780	4.01		S	Prophage endopeptidase tail		
	fig|1639.4014.peg.1788	3.89		S	Phage minor capsid protein 2		
	fig|1639.4014.peg.1793	3.87		S	Minor capsid protein		
	fig|1639.4014.peg.1805	3.8		S	Protein gp22 [Bacteriophage A118]		
	fig|1639.4014.peg.2062	3.61		S	Phage tail tape measure protein		
	fig|1639.4014.peg.2783	3.41		S	COG5546 Small integral membrane protein		
	fig|1639.4014.peg.1787	3.34		S	Phage portal protein, SPP1 Gp6-like [Bacteriophage A118]		
	fig|1639.4014.peg.1759	6.2		S	Protein gp44 [Bacteriophage A118]		
	fig|1639.4014.peg.468	5.43		V	Type VII secretion protein EsaA		
**Amino sugar and nucleotide sugar metabolism**							
lmo0957	fig|1639.4014.peg.2911	3.89	nagB	G	Glucosamine-6-phosphate deaminase	3.5.99.6	7453 48 h
lmo0956	fig|1639.4014.peg.2912	3.55	nagA	G	N-acetylglucosamine-6-phosphate deacetylase	3.5.1.25	
lmo0096	fig|1639.4014.peg.505	3.03		G	PTS system mannost-specific transporter subunits IIAB	2.7.1.191	
lmo0097	fig|1639.4014.peg.506	2.99		G	PTS system mannose-specific IIC component		
Lmo0783	fig|1639.4014.peg.1297	4.18	manX	G	PTS system mannose-specific IIAB component	2.7.1.191	
lmo2552	fig|1639.4014.peg.192	3.7	murZ	M	UDP-N-acetylglucosamine 1-carboxyvinyltransferase	2.5.1.7	
**Ribosome**							
lmo1542	fig|1639.4024.peg.1293	3.82	rplU	J	LSU ribosomal protein L21p		7545 48 h
lmo0250	fig|1639.4024.peg.1620	3.36	rplJ	J	LSU ribosomal protein L10p (P0)		
lmo2629	fig|1639.4024.peg.266	4.02	rplB	J	LSU ribosomal protein L2p (L8e)		
lmo2622	fig|1639.4024.peg.273	3.85	rplN	J	LSU ribosomal protein L14p (L23e)		
lmo2618	fig|1639.4024.peg.277	4.56	rpsH	J	SSU ribosomal protein S8p (S15Ae)		
lmo2617	fig|1639.4024.peg.278	4.01	rplF	J	LSU ribosomal protein L6p (L9e)		
lmo2616	fig|1639.4024.peg.279	3.85	rplR	J	LSU ribosomal protein L18p (L5e)		
lmo2613	fig|1639.4024.peg.282	3.57	rplO	J	LSU ribosomal protein L15p (L27Ae)		
lmo0044	fig|1639.4024.peg.808	4.46	rpsF	J	SSU ribosomal protein S6p		

*FDR < 0.01 log_2_ fold change.

^#^Time point.

**Table 3:** ST101 pathways overexpressed in differentially* expressed genes at 24 and 48 h in *L. monocytogenes*.

Locus tag (EGD-e)	Locus tag (this study)	FC	Gene	COG Cat	Gene/protein name	KEGG	Isolate and TP^#^
**Prophage related genes**							
	fig|1639.4037.peg.3124	9.61		S	Microvirus J protein; Phage DNA binding protein		ST101 24 h up regulated
	fig|1639.4037.peg.3125	10.88		S	Bacteriophage scaffolding protein D		
	fig|1639.4037.peg.3120	10.95		S	Bacteriophage replication gene A protein (GPA)		
	fig|1639.4037.peg.3126	10.99		S	Phage protein C; Phage single stranded DNA synthesis		
	fig|1639.4037.peg.3123	11.06		S	Capsid protein (F protein); Phage major capsid protein		
	fig|1639.4037.peg.3121	11.21		S	Microvirus H protein (pilot protein); Phage minor capsid protein		
	fig|1639.4037.peg.3122	11.83		S	Major spike protein (G protein)		
	fig|1639.4037.peg.3127	12.1		S	Bacteriophage replication gene A protein (GPA)		
**Pyrimidine metabolism and Alanine, aspartate and glutamate metabolism**							
lmo1838	fig|1639.4037.peg.1939	− 4.37	pyrB	F	Aspartate carbamoyltransferase	2.1.3.2	ST101 48 h down regulated
lmo1837	fig|1639.4037.peg.1938	− 4.32	pyrC	F	Dihydroorotase	3.5.2.3	
lmo1036	fig|1639.4037.peg.1937	− 4.2	pyrAa	F	Carbamoyl-phosphate synthase small chain	6.3.5.5	

*FDR < 0.01 log2 fold change.

^#^Time point.

#### Differential expression of select regulator genes

Seven of the regulatory genes selected for their association with stress response were significantly differentially expressed (DE) across three of the isolates (Table 4). Three regulatory genes, *fur*, *lexA* and *recA* were DE in isolate 7453 at 48 h with the logFC range between 2.56 and 4.16. Isolate 7538 displayed DE of four genes, *ctsR*, *degU*, *recA* and *sigB* at 48 h with the logFC ranging from 3.22 to 3.81. The *mogR* gene (logFC 1.67) and *recA* (logFC 2.32) gene was DE in isolate 7545 at 24 h and 48 h respectively. All other time points and isolates were negative for significant differential expression of the selected regulatory genes. Interestingly, *recA* was the only regulatory gene which was DE across three different isolates all at the 48 h timepoint and a logFC range from 2.32 to 3.42.

**Table 4:** Fold change of regulator genes differentially expressed at FDR < 0.05.

	7453^F^		7538^S^		7545^F^	
	24 h	48 h	24 h	48 h	24 h	48 h
*ctsR*				3.61		
*degU*				3.22		
*fur*		4.16				
*lexA*		2.82				
*mogR*					1.67	
*recA*		2.56		3.42		2.32
*sigB*				3.81		

## Discussion

*Listeria monocytogenes*’ ability to colonise FPE is a concern for the health of the at-risk population and the processing facilities’ economic viability and reputation. A deeper knowledge of *L. monocytogenes*’ ability to colonise and survive in FPE is required. The ability to replicate conditions representative of the FPE will assist in improving our understanding of these dynamics, however there are multiple complex elements involved including the type of contact surfaces (food and non-food) present, temperature, time, nutrients and ability to form biofilms. The availability and type of nutrients varies depending on the type of food or food products processed. While it is difficult to replicate the exact nutritional content available in the FPE, it is known to alternate between high and low nutritional stages during cycles of production. In this study, we assessed colonization behaviour of *L. monocytogenes*, incorporating these factors to reflect those of the FPE.

Initially there was some debate in the literature on *L. monocytogenes* ability to form biofilms, however there is growing evidence to suggest biofilm formation is a key component of the survival and persistence of some strains^16,20–22^. Early studies have tried to associate biofilm formation to a lineage or serotype with varying results^23–25^. In this study, biofilm formation was observed to be strain-specific as there was no consistency in the fast or slow biofilm groups linking a given phenotype to a specific genotype, as discussed below, and initially suggested in earlier *L. monocytogenes* biofilm studies^25–28^. Although two isolates belonging to MLST ST155 were present in the faster biofilm forming group, ST101 and ST2 had an isolate in both faster and slower groups, indicating no clear phenotype association with genetic sub-lineage. As attachment and biofilm formation appears to be environment and strain-specific we sought to determine what additional components may be of influence.

In FPE, access to nutrients can be transient therefore *L. monocytogenes* cells need to be able to adapt to the environmental conditions available. Biofilm formation studies have assessed the impact of nutrient deprivation, such as the study by Kadam et al.^25^ reporting enhanced biofilm formation and attachment was positively influenced in nutrient poor media. Cherifi et al.^29^ assessed BHI and a diluted BHI media with similar results. The results from this study correlate with *L. monocytogenes* ability to form biofilms in a low nutrient environment. This ability to adapt to low nutrient conditions may account for some of the differences in biofilm formation seen at 24 h, however by 96 h these variances were not apparent; this was also observed by Harvey et al.^30^, indicating initial attachment within 24–48 h is key to FPE colonisation.

A potential influence on attachment and biofilm formation during the first 24 h is the growth rate of isolates. While it is well known there can be differences in growth rate between strains, in this study the ability to form biofilms was not associated with growth rate and doubling times at 14 or 37 °C which reflects the results of other published research. The independent nature of biofilm formation to growth rate has been reported in previous studies at temperatures reflecting FPE and also at 37 °C^24,31^. Lee et al.^32^ noted less biomass was produced at 10 °C compared to biofilms at 37 °C, which were attributed to a lower growth rate and cell hydrophobicity at the cold temperature. Taylor and Stasiewicz^33^ also found persistent strains did not display increased ability to grow in various energy sources and conditions with their ability to persist most likely strain-specific or the result of environmental conditions.

The extracellular matrix (ECM) is a necessary component of the biofilm structure and is composed of proteins, extracellular DNA, polysaccharides and exopolysaccharides and amyloid fibres, however the composition varies between species^34^. While *Listeria* is not known to be a producer of cellulose and poly-β-1,6-*N*-acetyl-d-glucosamine common amongst Proteobacteria which produce defined biofilms, it has been reported *Listeria* produces a novel EPS primarily composed of *N*-acetylmannosamine and galactose which is capable of binding congo red as an indicator^35,36^. Two phenotypes were present in the B10 group, pink indicative of some EPS production and translucent, negative for EPS production, with the production depended on the medium used for some strains. While the amount of EPS produced was not determined, the presence of EPS in *Listeria* has been linked with cell aggregation and increased tolerance to disinfectants and desiccation suggesting the B10 strains which are capable of producing EPS have increased ability to survive and persist within the FPE and display initial stages of biofilm formation^35^. EPS production was not associated exclusively with either faster or slower biofilm formation.

The *agr* system was initially described as a signalling peptide system in staphylococcal species^37^, with orthologs *lam*^38^ and *fsr*^39^ being identified in *Lactobacillus plantarum* and *Enterococcus faecalis*, respectively, in addition to *L. monocytogenes*. The *agr* system is a peptide signalling communication four gene operon composed of *agrB*, a transmembrane protein which processes the propeptide encoded by *agrD* into a mature autoinducing peptide (AIP). The AIP is then exported into the extracellular environment until the concentration achieves a certain threshold, triggering the histidine kinase sensor *agrC* and activating the response regulator *agrA* which combine as a two-component system (*agrC-agrA*) applying transcriptional regulation including positive regulation^40–44^. The *agr* system has been shown in *L. monocytogenes* to be involved in invasion, pathogenicity and biofilm formation^45^. While this system has been shown to be linked to biofilm formation there is limited research on differences in expression between strong and poor biofilm producers at conditions reflecting the FPE. In this study, there was some statistical differences when comparing cells isolated from coupons to SM within either the fast or slow group; however, there was no statistical difference in the expression of *agrD* between the fast isolates and the slow isolates. Gandra et al.^46^ reported higher levels of the *agr* locus is expressed at 37 °C compared to 10 °C. In addition, they identified *agrBCD* genes are important for adhesion and the initial stages of biofilm formation particularly at 12 and 24 h. The results of this study support the upregulation of *agr* system elements in the early stages of attachment and early biofilm growth; however, expression appears to decline as the biofilm matures. In contrast, increased *agrD* expression was not observed in the planktonic cells of the spent media in this study at any of the timepoints measured, suggesting expression of this signal peptide is induced following attachment and initial biofilm formation, rather than planktonic growth, under the conditions tested.

To further investigate a genetic basis for the rapid colonisation phenotype, this study also examined the global transcriptomic response of *L. monocytogenes* during attachment and biofilm formation at 24 and 48 h under conditions reflective of the FPE. Four isolates from two STs (two isolates per ST) were chosen for RNA sequencing, with each ST cohort including one fast and one slow coloniser, to provide insights into variation in gene expression between fast and slow colonisation phenotypes. This included a lineage I and lineage II ST. Globally across strains metabolism and transport pathways were up regulated with variation of the pathways between strains. As a saprophyte, *L. monocytogenes* is exposed to varied, and at times limited nutrient sources and as such requires an extensive range of transport and metabolism mechanisms. Glaser et al.^47^ identified 331 different transporter genes with 88 related to the phosphoenolpyruvate-dependent phosphotransferase systems (PTS) responsible for the transport and phosphorylation of various sugars and sugar derivates^48^. This extensive range of transporter genes is one of the largest known among bacterial species and allows *L. monocytogenes* to survive within a broad range of environmental and host conditions^47^. Furthermore, it allows for the bacterium to respond to any changes in its environment and adapt as necessary. In a few other bacterial species in which the biofilm genetic landscape has been eluded, PTS has been linked with the regulation of biofilm formation. In a study on *Klebsiella pneumoniae* biofilms, three genes encoding an enzyme II complex in PTS was found to increase eDNA and capsular polysaccharide production resulting in positive regulation of biofilm production^49^. Similarly, Houot and Watnick^50^ found the *Vibrio* polysaccharide (*vps*) genes of *Vibrio cholerae* responsible for exopolysaccharide synthesis, were coregulated with PTS components and formation of multilayer biofilms were influenced by particular PTS sugars which activated the transcription of these *vps* genes. Unlike *V. cholerae*, the genetic determinants for *L. monocytogenes* biofilms are not well defined and comprise of a variety of genetic interactions, with most also having an established role in virulence and pathogenicity. In our study, various components of the PTS were upregulated at 24 h across the fast isolates, compared to the slow isolates, however there is limited research assessing how the PTS influences biofilm formation at conditions reflective of the FPE in *L. monocytogenes*. In this study, various elements of the PTS pathways up regulated in different strains further suggests colonisation differences are strain-specific and influenced by environmental conditions. Further research is required to determine if various components of the PTS are responding to its preferred nutrients as the result of the isolation environments selected in this study, or if the PTS have roles in the early stages of biofilm formation.

In conjunction with the PTS, various metabolic pathways associated with carbohydrates and sugars were also upregulated, including starch and sucrose metabolism at 24 h and amino and nucleotide sugar metabolism at 48 h across the fast isolates suggesting a switch to nutrient scavenging to initiate colonisation. Free glucose is often not readily available in the environment and as such alternative carbon sources are required. As mentioned previously, *L. monocytogenes* has an extensive transport system allowing this bacterium the ability to utilise various environmental carbon sources at times when nutrients are limited. Energy sources like cellobiose, lactose, lichenan, trehalose, maltose and their associated degradation products were all up regulated in this study as well as the 6-phospho-β-glucosidase, which suggests beta-glucosides are used by these strains. Taylor and Stasiewicz^33^ found 97% of *L. monocytogenes* isolates tested (n = 95) grew in defined media supplemented with cellobiose, fructose or glucose however, lactose and sucrose were unable to support the growth of 79 and 72% of the isolates, respectively. An earlier study also reported fructose, mannose, cellobiose, trehalose were capable of supporting *L. monocytogenes* growth in the absence of glucose^51^. Mannose and trehalose supplementation has also been shown to increase biofilm development over 12 days^52^. The results of this study suggest a global upregulation of diverse metabolic pathways under nutrient limited, low temperature conditions may facilitate adaptation and maximised nutrient scavenging, contributing to initiation of a biofilm lifestyle and persistence of *L. monocytogenes* under similar conditions founds in FPE.

Amino sugar metabolism has been connected to energy production and biosynthesis of cell wall peptidoglycan and teichoic acids^53,54^. Key enzymes of the amino sugar and nucleotide sugar pathway up regulated in this study at 48 h includes *N*-acetylglucosamine-6-phosphate deacetylase (*nagA*) and glucosamine-6-phosphate deaminase (*nagB*), indicating at 48 h under conditions reflective of the FPE the fast isolates are undergoing an increase in biomass through the biosynthesis of peptidoglycan cementing their ability to survive in the FPE. N-Acetylglucosamine (GlcNAc) is an abundant carbon and nitrogen source found throughout the environment (as a chitin monomer) and as part of bacterial cell wall peptidoglycan^55^; it has been reported *L. monocytogenes* can turnover between 30–50% of its cell wall peptidoglycan every generation^53^. The deacetylation of N-acetyl-glucosamine-6-phosphate by NagA into glucosamine-6-phosphate and acetate is a part of peptidoglycan degradation and thus cell wall recycling^53^. Glucosamine-6-phosphate can be further transformed into fructose-6-P by NagB for energy production through the glycolysis pathway^54^. An additional key enzyme in peptidoglycan biosynthesis is UDP–*N*-acetylglucosamine (UDP-GlcNAc) 1-carboxyvinyltransferase (MurA) responsible for the addition of enolpyruvyl from phosphoenolpyruvate to UDP-GlcNAc^56^. The paralogue version, *murZ* was up regulated in this study. The combination of *nagA*, *nagB* and *murZ* suggests cells were possibly undergoing cell wall synthesis to increase biofilm mass. This adaptation again suggests a global switch to nutrient scavenging and biomass increase is a central strategy to the initial colonisation of FPE by *L. monocytogenes*.

Three genes involved in pyrimidine metabolism and alanine, aspartate and glutamate metabolism pathways were upregulated at 48 h in the ST101 comparison. The genes observed related to pyrimidine metabolism are involved in de novo synthesis of uridine-monophosphate (UMP) starting from glutamine and include, *pyrAa*, carbamoyl-phosphate synthase small chain, glutamine-utilizing subunit of carbamoyl-phosphate synthetase, similar to the *carA* of the same role in *E. coli*, *pyrB*, catalytic subunit of aspartate carbamoyltransferase and *pyrC*, dihydroorotase^57,58^. A study by Pisithkul^59^ into biofilm development of *Bacillus subtilis* found expression of pyrimidine synthesis enzymes and other nucleotides and biosynthetic precursors peaked at 16 h then declined slowly for the remainder of the study. In another study, Hingston^60^ identified *pyrAaBC* genes were up regulated at 4 °C during the transition to stationary phase. De novo synthesis of UMP has been linked to biofilm formation and production of cellulose and curli fimbriae in *E. coli* through transcription of the *csgDEFG* operon^61^. While the *pyr* operon has not been linked to biofilm formation in *L. monocytogenes*, our results suggest it may be linked in some way, however further research is required.

Interestingly, the cobalamin biosynthesis pathway and genes involved in the cobalamin-dependent gene cluster (CDGC) were also identified as being overexpressed at 24 h in isolate 7453^F^. Cobalamin genes are responsible for vitamin B_12_ biosynthesis which is required as enzyme cofactors for various metabolic process particularly during the metabolism of ethanolamine and 1,2-propanediol as carbon and nitrogen energy sources^62^. Cobalamin biosynthesis can occur during aerobic respiration with *cob* genes or during anaerobic respiration utilising *cbi* genes^63^. In this study more *cbi* genes (compared to *cob*) from the Cobalamin anaerobic pathway were upregulated, in addition, genes involved in ethanolamine (FC range 3.2–6.8) and propanediol utilisation (FC range 2.03–4.2) were also up regulated and have been shown to be activated during stressful, competitive conditions and during cold temperatures^64–67^. In a transcriptomic study by Hingston and colleagues^60^, they reported an increase in genes associated with ethanolamine utilisation at multiple growth phases at 4 °C. The upregulation of the genes from the CDGC may reflect *L. monocytogenes* is experiencing stress as a result of the low temperature and limited nutrients within the biofilm state. These systems facilitate greater flexibility in nutrient scavenging and utilisation through metabolism of alternative substrates, which is critical for survival when optimal nutrients are unavailable or competition with other microbial species is ongoing^66,68^.

Ribosomes are essential protein synthesising components that are involved in sensing and responding to their environmental conditions^69^. In prokaryotes they are composed of a 50S large subunit, where the peptide bonds are formed, and a 30S small subunit that binds the messenger RNA, creating a 70S ribosome^70^. In this study, a variety of ribosomal proteins were upregulated with a majority being the large subunit. Each subunit contains 30 and 20 ribosomal proteins (R-proteins) designated L or S for the 50S or 30S subunits respectively. R-proteins have various roles including translation, assembly, cell proliferation and cellular differentiation with some of these roles essential for survival^71^. In this study, up regulation of ribosome proteins may reflect the global level of transcription and translation is higher under conditions reflective of the FPE due to multiple sub-optimal factors at play, however, there is limited research on the R-proteins in *L. monocytogenes* to be able to elude to more specific roles in this study.

Within isolate 7453^F^ and ST101 at 24 h there was a considerable number of differentially expressed prophage genes expressed suggesting prophage genes may influence the initial stages of colonisation. Over 500 *L. monocytogenes* bacteriophages have been identified, with a large portion being temperate phages capable of inserting themselves into the bacterial chromosome^72^. Temperate phages have been linked with providing increased fitness to host bacterial strains^73^. A common temperate listeria phage A118 has been shown to insert itself into the competence protein K (*comK*). A study by Verghese et al.^28^ showed meat and poultry isolates containing the *comK* prophage were capable of growing to higher cell densities with the authors suggesting its insertion allows strains to adapt to niches which influence their colonisation and persistence in FPE. In an earlier study on *E. coli* K12 strains containing cryptic prophage, they found increased fitness against osmotic, oxidative and acidic stress and increases in biofilm formation and growth^73^. While there have been limited studies reporting lab based phenotypic benefits of *L. monocytogenes* isolates containing prophages, the up regulation of prophage genes in this study opens the possibility they may play some role in either low nutrient adaption, attachment or biofilm formation. In this study, phage A118 is inserted into the *comK* gene of isolate 7453^F^ suggesting the presence of phage A118 may influence this isolate’s ability to rapidly colonise the FPE by increasing cell density and withstanding the suboptimal conditions found in FPE.

The DE of regulators and repressors involved in stress response and biofilm formation can be indicators of which stress systems are responding to sub-optimal conditions, it is important however to note that it is not one particular regulator being induced rather a variety of different regulators and repressors indicating the complex nature of the FPE and the overlap in stress response and virulence related genes and systems. In this study, *recA/lexA*, responsible for DNA repair and activation of the SOS response during stressful conditions in *L. monocytogenes*, was upregulated in three strains and one strain respectively. The SOS response is required for bacterial adaptation, diversification and pathogenesis in a majority of species and has been reported to be required for biofilm formation in *E. coli*, *Pseudomonas aeruginosa*, *Staphylococcus aureus*, and *Mycobacterium tuberculosis*^74^. Van der Veen et al.^75^ showed *recA* also influences genetic variability through mutagenic repair during continuous flow biofilms. The mutagenic repair of DNA may be critical for biofilm formation and resistance to stress conditions along with the development of disinfectants and antibiotic resistance^76^. The upregulation of *recA* and *lexA* is indicative of the stress conditions experienced in this study from the low temperature and limited nutrients utilised.

The presence of flagella and motility related genes have been shown to be involved in initial attachment stages and subsequent biofilm formation and colonisation in *L. monocytogenes* on various processing environment and produce surfaces^77,78^. In isolate 7538 the *degU* response regulator was upregulated. Previously, *degU* has been associated with flagella biosynthesis, chemotaxis, attachment and biofilm formation^79,80^. Gueriri et al.^80^ suggests that *degU* may play a role in biofilm formation that is distinct from the essential role it plays in regulating flagella synthesis. In addition, Pieta et al.^81^ showed *degU* was equally or significantly increased at 7 °C when compared with 37 °C. Therefore, the upregulation of *degU* is suggestive of cells undergoing biofilm formation, as strain 7538 was a slow biofilm former it may be the motility of cells at lower temperatures may be regulated later compared to fast biofilm formers.

The *mogR* gene is the transcriptional repressor of flagella motility at all temperatures and specifically at temperatures relevant for infection^82^. Cordero et al.^83^ reported strains which demonstrated faster growth rates at low temperatures displayed reduced flagella expression to conserve energy yet remain prolific. Isolate 7545 displayed *mogR* expression suggesting flagella motility was reduced potentially as a metabolic function to save energy and continue multiplying in the limited nutrients and low temperature conditions used in this study.

The class three stress gene repressor, *ctsR*, which regulates class three heat shock genes was upregulated in isolate 7538. In addition, *ctsR* has also been indirectly linked with virulence, motility gene expression and has been shown to be coregulated with other regulators including *sigB*, *sigH*, *hrcA* and *prfA*^60,84–86^. The general stress response gene, *sigB*, was also upregulated in this isolate. The alternative sigma factor β (*sigB*) is a major stress response regulator of general stress response and class II stress genes which are required for various stress related conditions, including cold, acidic, osmotic, oxidative stress and high pressure processing^87,88^. *SigB* has been shown to be required for the biofilm mode of life in both, static and continuous flow biofilms^87^, in addition there are reports it is required during starvation survival in low nutrient environments^89^. In this study, suboptimal conditions used were to reflect the stressful climate in the FPE therefore the upregulation of *ctsR* and *sigB* is an adaptive mechanism *L. monocytogenes* most likely employees to survive within the FPE.

The *fur* gene is required to regulate intracellular levels of iron which is an essential cofactor required for many important enzymatic roles in bacterial cells^90,91^. *Fur* regulation has been linked with oxidative stress response and protection against ROS damage^92,93^. In addition, in low iron environments f*ur* regulation plays a significant role in sequestering iron within increased levels of *fur* transcription reported in these environments^92,94^. The upregulation of f*ur* may be indicative of low iron levels as a result of the limited nutrient environment or cold stress conditions utilised in this study. Further, *fur* regulation has been linked with metabolic function in bacteria^94^; the conditions in this study resulted in a diverse range of metabolic systems upregulated and therefore the upregulation of *fur* may be reflective of the metabolic state cells in biofilm are undergoing.

In this study we aimed to replicate elements present in the FPE to determine their influence on the colonisation by *L. monocytogenes*. Although the results obtained provide beneficial insight into our understanding of this subject, it was not without its drawbacks. The multiple factors analysed in combination have provided some generalised understanding and identified baseline research against more isolates is required. For the ST comparison the isolates were not isogenic strains however based upon the average nucleotide identity (99.91% for isolates 7453 and 8116 and 99.90% for isolates 7545 and 7538) the isolates selected were considered suitable for comparison purposes. In addition, the expression data for a small number of genes which are not shared between the comparison isolates may be absent as a result of using non-isogenic strains.

## Concluding remarks

*L. monocytogenes* isolates are a concern for public health due to their ability to colonise and persist in FPEs. The economic and brand reputation for a food processing company can be substantial should *L. monocytogenes* strains contaminate RTE food products and cause listeriosis. This study looked at various factors which may influence *L. monocytogenes* ability to colonise a processing facility. We demonstrated that the ability to form biofilms was different from strain to strain and was not linked to differences in growth at conditions reflective of the FPE, nor cellulose or curli expression as identified in other species like *E. coli* and *Salmonella*. While there were also no specific genes identified by the GWAS, interestingly the global transcriptome indicated metabolic mechanisms were upregulated, suggesting the species utilizes its wide metabolic and transport repertoire to initiate a rapid adaptation to nutrient limited conditions. This is then coupled with upregulation of genes involved in the production of cell structural components for biofilm expansion, with upregulation of the *agr* system in the initial attachment and biofilm growth. Colonisation is likely aided through environmental factors like hard to clean and sanitise niches, and genetic determinants like the ability to form biofilms and attach in suboptimal conditions, our knowledge of *L. monocytogenes*’ ability to persist and survive in the FPE requires further exploration, as this knowledge will be necessary in order to prevent and mitigate contamination.

## Methods

### Bacterial isolates, culturing conditions and subtyping

A total of 52 *L. monocytogenes* isolates from 12 sequence types (ST, up to 5 isolates per ST) representative of multi-locus sequence types commonly associated with FPEs in previous analyses^95,96^, and previously isolated from a variety of food-related sources (i.e., dairy, meat, vegetable, mixed food and environment; Supplementary Table S1) were chosen. Isolates selected each possessed unique pulsed field electrophoresis pulsotypes, to increase strain variance. Isolates were removed from − 80 °C storage and resuscitated on Brain Heart Infusion (BHI, CM1136, Oxoid, UK) agar at 37 °C for 24 h.

### Stainless steel coupons

Stainless steel (SS) coupons of grade 304, mill finish (5 mm diameter by 0.9 mm thick; surface area 0.53 cm^2^) were utilized. Coupons were cleaned in a solution of 3% sodium hydroxide (Sigma-Aldrich, 72068, Australia) for 20 min, then 0.1% peracetic acid (Oxysan, C16620, Australia) for two minutes. Coupons were rinsed with sterile water three times between washes and then sterilised in the autoclave.

### Biofilm formation analysis

*L. monocytogenes* isolates were grown for 18 h (± 1 h) in BHIB at 37 °C. A high throughput biofilm screening method, previously developed^97^, was used to determine the fastest and slowest biofilm forming isolates. Briefly, microtiter plate wells containing SS coupons were inoculated aseptically with 100 µL of 10^3^ CFU/mL in 1:10 diluted BHI (dBHI) and incubated at 14 °C for 24, 48, 72 or 96 h (± 1 h) statically. After the appropriate incubation period the spent medium were removed, SS coupons were transferred to a sterile microtiter plate and underwent three rinses with sterile water. Coupons were sonicated in wells with Maximum Recovery Diluent (MRD; Oxoid, Thermo Scientific, Australia) for 5 min then 100 µL was serial diluted and plated onto BHI agar (BHIA) for enumeration at 37 °C for 24 h prior to counting. Two biological replicates each with two technical replicates were performed on all 52 isolates, with an additional two biological replicates, again with two technical replicates, performed on 10 isolates. These 10 isolates comprised those with the fastest (*n* = 5) or slowest average biofilm cell numbers after 24 h (referred to as the B10 isolates).

### Growth rate determination

Growth curves were constructed for the planktonic B10 isolates at 37 °C in undiluted BHI and at 14 °C in dBHI. For the growth curves, a single colony of each B10 isolate was inoculated in 5 mL BHI at 37 °C at 150 rpm for 18 h (± 1 h). For the 37 °C growth curve, 200 µL of a 1:200 dilution was aliquoted into a 96 well microtiter plate and growth was monitored for 12 h at OD_600_ using an EON microplate spectrophotometer Gen5 (BioTek, Australia). For the 14 °C growth curves, a 1:200 dilution of the 18 h (± 1 h) culture into dBHI was aliquoted into conical flasks and growth was monitored every 4 h until timepoint 15 h when growth was measured every 2 h at OD_600_ for 31 h. Maximum growth rate (µ) and doubling times (t_d_) (2) were determined during the exponential growth phase using the equations: µ = (ln OD_2_ − ln OD_1_)/(t_2_ − t_1_) and t_d_ = 0.693/µ, respectively, where ln refers to the natural logarithm, OD_2_ is late exponential phase OD, OD_1_ is early exponential phase, t_2_ is time in minutes for OD_2_ reading and t_1_ is time in minutes for OD_1_ reading, t_d_ is doubling time and µ is growth rate.

### EPS production

Exopolymeric substance analysis was performed as follows: lysogeny broth (LB) agar without salt supplemented with 40 µg/mL Congo Red (CR) and 20 µg/mL Coomassie Brilliant Blue (CBB) was spotted with 5 µL of the 18 h (± 1 h) culture and incubated at 14 °C for 48–72 h and 37 °C for 24–48 h. For the CR assay, 18 h (± 1 h) cultures were grown in LB without salt and Muller Hinton broth at 37 °C and 150 rpm.

### Large batch biofilm formation

The biofilm process was upscaled for RNA extractions at 24 and 48 h for the B10 isolates. The biofilm process followed the initial screening experiment with the following changes: two coupons (15 mm × 15 mm × 0.55 mm) were used per isolate time point, coupons were transferred to a new 70 mL yellow cap container for three washes with DEPC-treated molecular grade water prior to biofilm removal with a cell scraper then sonication for 5 min. Cell scrapers were vortexed briefly for 10 s then pulse vortexed five times to remove any attached cells. Cells were pelleted at 7000×*g* for 10 min.

### Total RNA extraction

Total RNA was extracted using the RNeasy Mini Kit (Qiagen, Australia) with the following adjustments: 25 mL of spent medium (SM) was collected from each time point and 1 mL of *Escherichia coli* DH5α was added as a carrier to assist in centrifugation of pellet. For the coupons, 2 mL of *E. coli* DH5α was used. RNA stabilisation was performing using a 5% phenol ethanol solution as per Bhagwat et al.^98^. Enzymatic lysis of cells consisted of 100 units of mutanolysin (Sigma-Aldrich/Merck Australia) for 15 min in a 22 °C water bath followed by 20 µL of 20 mg/mL Proteinase K for an additional 15 min. During RNA purification the spin column was washed twice with Buffer RW1. RNA yields were assessed on a Nanodrop device ND-1000 (Nanodrop, Thermo Fisher, Australia) and RNA quality assessment was performed on 2200 TapeStation System (Agilent, Australia) using high sensitivity RNA screen tapes. Samples were stored at − 80 °C until reverse transcriptase and RNA sequencing.

### Genome wide association study

A genome wide association study (GWAS) was performed using the R package treeWAS^99^ to identify genetic variants potentially responsible for variances in the biofilm phenotype at 24 h utilising a phylogenetic method accounting for population structure and recombination. Kchooser and Ksnp3^100^ was used to generate the optimal kmer value and core SNP matrix file from the biofilm isolates genome sequences.

### Real time qPCR

DNase treatment and cDNA synthesis were performed on 1 µg of RNA using the iScript gDNA clear cDNA synthesis kit (Bio-rad, Australia) as per manufacturer’s protocol. Real time qPCR (RTi-qPCR) was performed targeting the propeptide *agrD* as the gene of interest and *rpoB* as the housekeeping normalisation gene on the AriaMX Real-time PCR System (Aglient). Primer sequences were designed using primer3 in Geneious (2018) (Table 5). RTi-qPCR amplification was performed in 20 µL reactions with the mix containing 10 µL iTaq universal SYBR green Supermix (Bio-rad), 1 µL forward and reverse primers, 6 µL molecular grade water (Sigma-Aldrich) and 2 µL cDNA. PCR conditions were as follows: 3 min at 95 °C followed by 40 cycles at 5 s at 95 °C and 45 s at 60 °C. Assays included a non-template control and non-reverse transcriptase for sample control with three biological replicates each with three technical replicates. Relative expression was determined using the 2^−∆∆^CT method^101^. Stata (Stata 15.1, StataCorp, College Station, Texas, USA) was used for statistical and data analysis. The nonparametric Wilcoxon rank sum test was performed on independent samples and the Wilcoxon signed rank test was performed on paired samples (p value < 0.05).

**Table 5:** Real time-PCR primer sequences designed for this study.

Primer set	Oligonucleotide sequence 5′ → 3′
agrD-F	CAGTTGGTAAATTCCTTTCTAGAAAAC
agrD-R	TTTTCACAAATGGACTTTTTGGTTCG
rpoB-F	TGGGGCAGAACGTGTTATCG
rpoB-R	CCCACGGTTAGGGATGACAG

### RNA sequencing and analysis/transcriptomics

Four isolates from two STs which had an isolate in both the fast and slow biofilm formation groups were chosen for RNA sequencing. Total RNA extracts for sequencing were measured using the Qubit RNA high sensitivity kit (Thermo Fisher) and RNA extracts were sequenced by Western Sydney University Next Generation sequencing facility (NSW, Australia). Zymo-Seq RiboFree Total RNA Library Prep kit was used for rRNA depletion following the manufacturer’s protocol. Depleted RNA samples were clustered on cBot and sequencing was performed as 2 × 125 bp paired end TrueSeq Cluster kit v4 and HiSeq SBS v4 kit on the Illumina HiSeq 2500 platform.

Sequence alignment was performed in Galaxy Australia^102^ using the following tools: reads were mapped to each isolates draft genome sequence using BWA-MEM (Galaxy v0.7.17.1)^103^, JBrowse genome browser was used to view the mapped reads (Galaxy v1.16.4 + galaxy3)^104^, SAM/BAM to count matrix using HTSeq code (v0.5) was used to produce differential gene expression (DGE) count matrices. Gffread (Galaxy v0.11.6.0) was used to convert .gff3 files from the Patric database^105,106^ to .gtf files for count analysis. The log_2_ counts per million for the DGE count matrix were determined by Voom/Limma in Degust (v4.1.1)^107^. Individual isolate comparisons consisted of 7453 24 h with 7453 48 h, 7545 24 h with 7545 48 h, 8116 24 h against 8116 48 h and 7538 24 h and 7538 48 h. For ST comparison, analysis was performed by comparing the two isolates from within the same ST at the same timepoint. The ST101 24 h comparison consisted of isolates 7453 24 h and 8116 at 24 h, the ST101 48 h comparison was against 7453 48 h and 8116 48 h. The ST2 24 h analysis was between 7545 24 h and 7538 24 h and the ST2 48 h comparison contained 7545 48 h and 7538 48 h. The draft genome sequences of 7538 and 8116 was used as the reference genome for ST2 and ST101 respectively. Functional annotation was performed with Eggnog mapper v2 (v2.0.0) using Listeriaceae as the taxonomic scope and gene ontology from experimental evidence only with all other fields default. The functional annotation was matched to differentially expressed genes (DEGs) using Excel and were analysed based upon their clusters of orthologous groups (COG) category with tRNAs allocated to COG category J and hypothetical proteins and DEGs with no COG category assigned to category S to include in the analysis. Overexpressed protein pathways were determined using STRING (v11)^108,109^ by submitting the amino acid sequences for all the DEGs (FDR < 0.01 and log_2_ FC) with *L. monocytogenes* EGD-e as the organism reference. Statistical significance was determined for overexpressed protein pathways with a false discovery rate (FDR) < 0.01 and absolute log fold change (logFC) of ≥ 2 for 24 h vs 48 h samples. Differentially expressed regulatory genes were determined utilising an FDR < 0.05. Regulatory genes of interested were determined based upon the conditions utilised in the simplified model biofilm system and reflected the isolates potential systems/pathways used to respond to these conditions and included the following genes: *ctsR, hcrA, lexA, perR, codY, agrA, sigB, fur, recA, mogR, degU, virR* and *prfA*.

## Supplementary Information

Supplementary Information.

Supplementary Table.

## Data Availability

The raw sequencing data were deposited at the NCBI Sequence Read Archive under Bioproject No. PRJNA715821.
