# Supplementary material for: Colonisation dynamics of Listeria monocytogenes strains isolated from food production environments
Source: Sci Rep. 2021 Jun 9;11:12195. doi: 10.1038/s41598-021-91503-w (PMC8190317; doi:10.1038/s41598-021-91503-w)
Supplement: Supplementary file 1 — Supplementary Information. [file 41598_2021_91503_MOESM1_ESM.pdf]

Supplementary Files

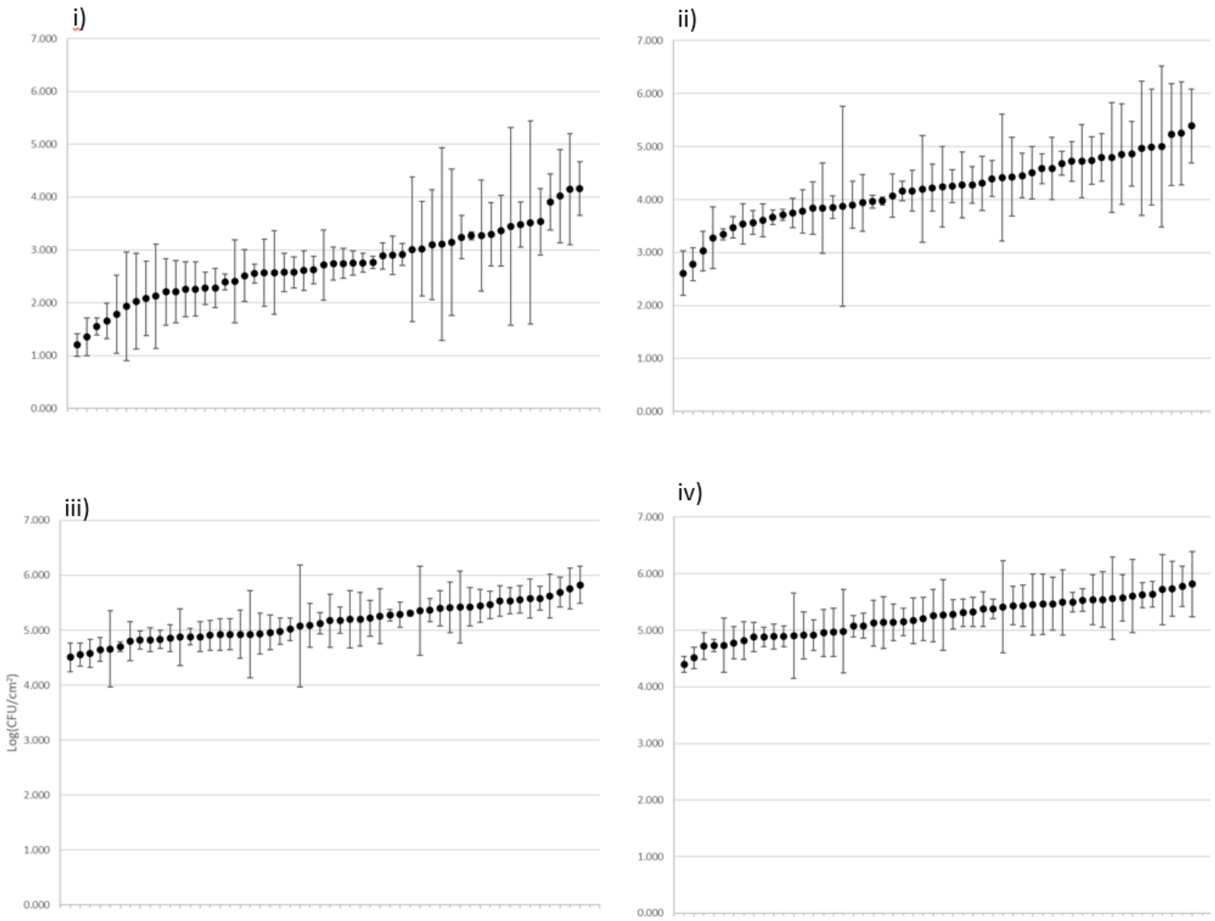

Supplementary Figure 1: Comparison of biofilm formation by 52 *L. monocytogenes* isolates at 14°C in dBHI on stainless coupons at i) 24 h, ii) 48 h, iii) 72 h and iv) 96 h. Biofilm densities (log_{10} CFU/cm^2) were determined every 24 h by standard plate count. Data points represent the average of two biological experiments with two technical replicates, with error bars showing standard deviation. All 52 isolates are displayed on the x axis in numerical order based upon average biofilm density from lowest to highest.

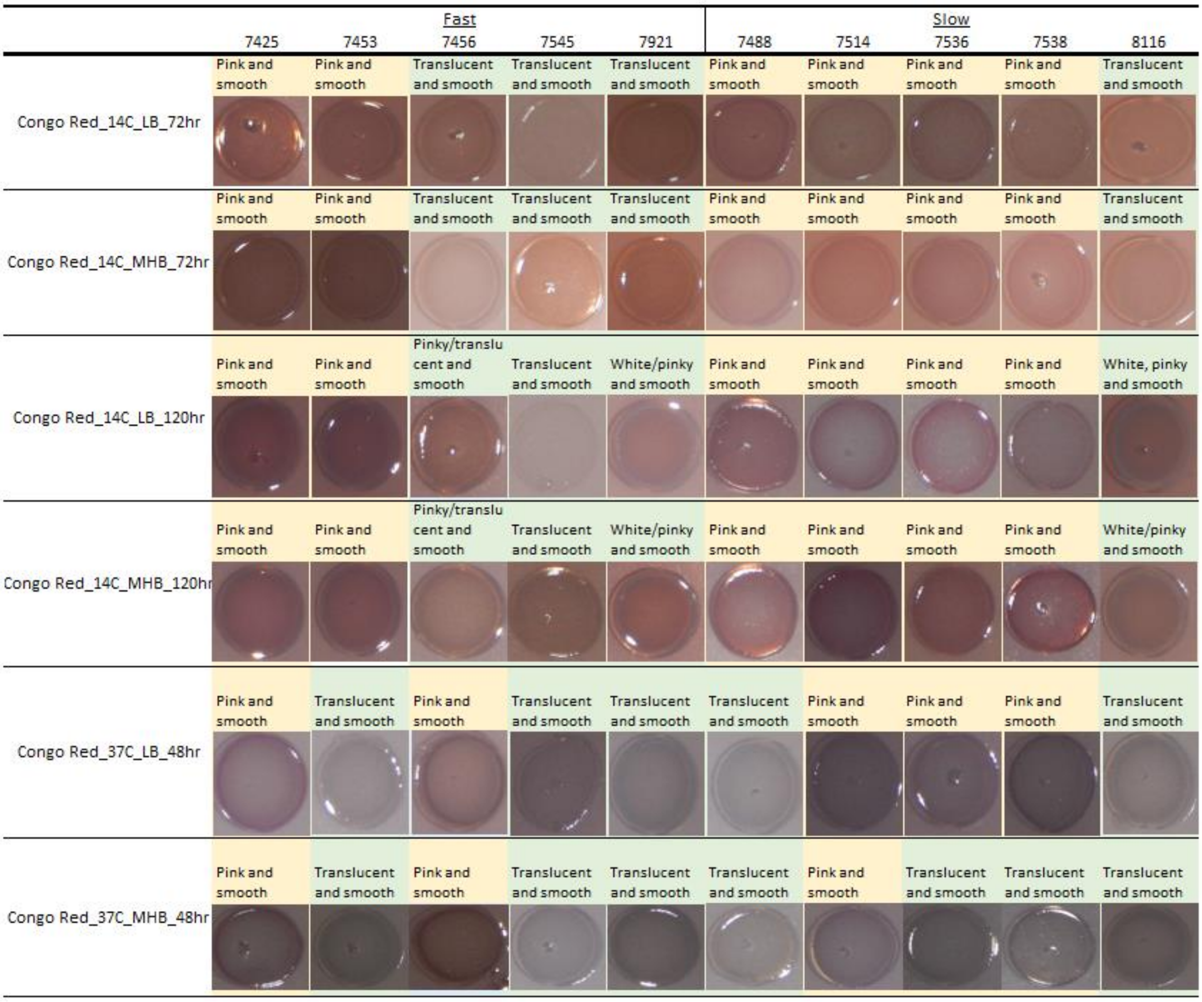

Supplementary Figure 2: EPS production by the B10 isolates on LB or Muller Hinton agar supplemented with 40 µg/mL Congo Red and 20 µg/mL Coomassie Brilliant Blue grown at 14 or 37°C for 48, 72 or 120 h. Pink phenotype indicative of intermediate ability to produce EPS and translucent phenotype suggestive of no EPS production.

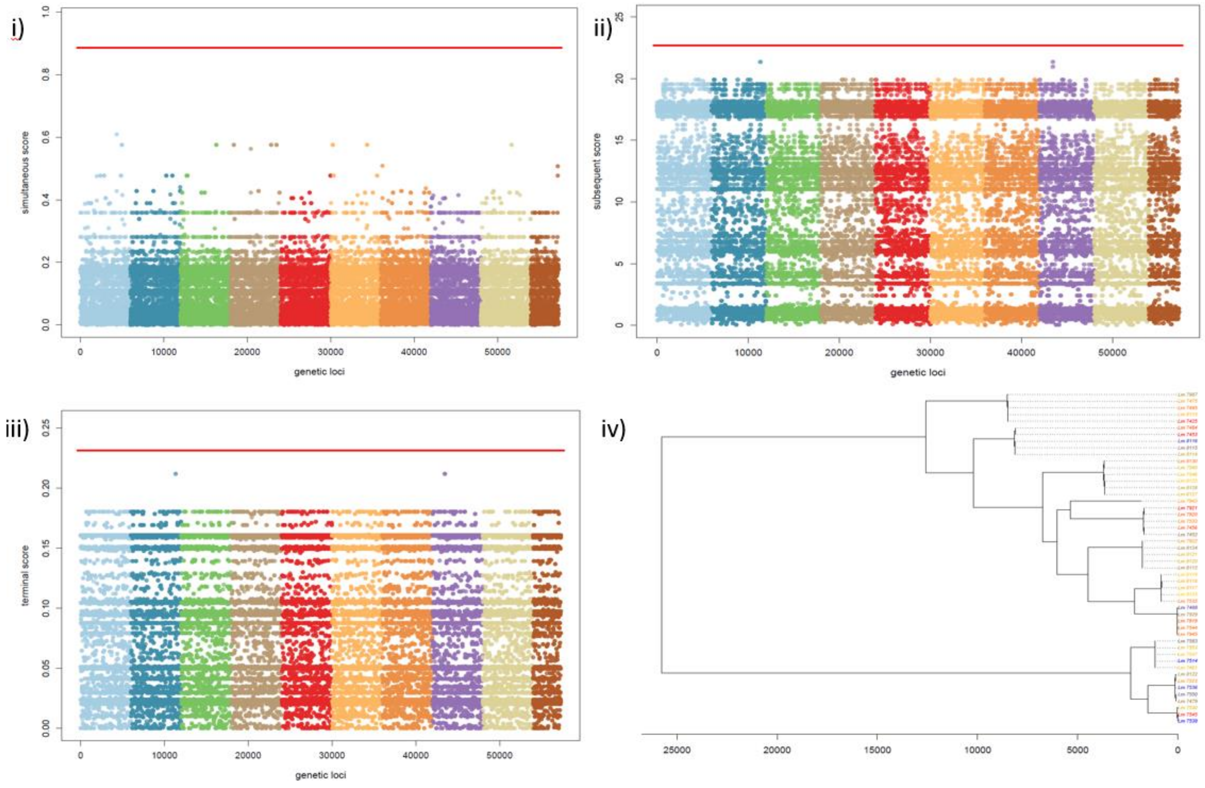

Supplementary Figure 3: treeWAS tests of association, red line is significance level. i) simultaneous score; ii) subsequent score; iii) terminal score; iv) phylogenetic tree based upon clonal complex, isolates label colours include blue (slow biofilm formers) and red (fast biofilm formers). treeWAS was performed using R software (v 3.4.1) [1].

Supplementary Table 1: Isolate information.

Isolate	MLST ST	Serotype	Year isolated	Description	Lineage	CC	Biofilm phenotype[#]	Genebank Accession #
7425	121	1/2a	2011	Environmental - Meat	II	CC121	Fast	SAMN17321006
7452	155	1/2a, 3a	2009	Dairy - Milk	II	CC155		NAUM00000000
7453	101	1/2a, 3a	2009	Dairy - Cheese	II	CC101	Fast	NAUH00000000
7456	155	1/2a, 3a	2010	Dairy - Milk	II	CC155	Fast	NAUL00000000
7461	3	1/2b, 3b	2010	Dairy - Milk	I	CC3		JAGVQS000000000
7475	121	1/2a, 3a	2011	Dairy - Cheese	II	CC121		NAUF00000000
7479	1	4b, 4d, 4e	2012	Dairy - Cheese	I	CC1		
7484	101	1/2a, 3a	2012	Dairy - Cheese	II	CC101		NAUG00000000
7488	204	1/2a, 3a	2012	Dairy - Cheese	II	CC204	Slow	LXQX00000000
7495	121	1/2a, 3a	2012	Dairy - Cheese	II	CC121		NAUE00000000
7514	3	1/2b, 3b	1998	Vegetable	I	CC3	Slow	JAGVQT000000000
7523	1	4b, 4d, 4e	1988	Meat - Beef	I	CC1		NAVA00000000
7530	2	4b, 4d, 4e	1998	Dairy - Ice cream	I	CC2		JAGVQU000000000
7533	155	1/2a	2007	Environmental - Meat	II	CC155		JAGVQV000000000
7535	9	1/2c	2007	Environmental - Meat	II	CC9		LJPE01000000
7536	1	4b, 4d, 4e	2009	Environmental - Dairy	I	CC1	Slow	LJPF01000000
7538	2	4b, 4d, 4e	2009	Environmental - Dairy	I	CC2	Slow	JAGVQW000000000
7540	7	1/2a, 3a	2011	Mixed Food	II	CC7		JAGVQX000000000
7544	204	1/2a, 3a	2006	Environmental - Dairy	II	CC204		LXQZ00000000
7545	2	4b, 4d, 4e	2008	Mixed Food	I	CC2	Fast	JAGVQY000000000

7546	12	1/2a, 3a	2009	Environmental - Dairy	II	CC7		JAGVQZ000000000
7547	3	1/2b, 3b	2009	Dairy - Cream	I	CC3		JAGVRA000000000
7550	1	4b, 4d, 4e	2013	Dairy - Cheese	I	CC1		
7553	3	1/2b	2007	Environmental - Meat	I	CC3		JAGVRB000000000
7583	3	1/2b, 3b	2007	Environmental - Dairy	I	CC3		NAUZ00000000
7919	204	1/2a	2015	Meat - Boots	II	CC204		LXRA00000000
7920	155	1/2a	2015	Meat - Boots	II	CC155		JAGVRC000000000
7921	155	1/2a	2015	Meat - Boots	II	CC155	Fast	JAGVRD000000000
7922	8	1/2a	2015	Meat - RTE	II	CC8		JAGVRE000000000
7929	204	1/2a, 3a	2015	Meat - Raw Ingredient	II	CC204		LXRB00000000
7943	321	1/2a, 3a	2015	Meat - RTE	II	CC321		JAGVRF000000000
7945	204	1/2a or 3a	2015	Meat - RTE	II	CC204		LXRC00000000
7987	121	1/2a	2016	Meat	II	CC121		JAGVRG000000000
8112	8	1/2a,3a	2010	Meat	II	CC8		SRR6457844
8113	121	1/2a,3a	2009	Environment - Equipment	II	CC121		SRR6457840
8114	101	1/2a,3a	2009	Vegetable	II	CC101		SRR6457839
8115	101	1/2a,3a	2009	Vegetable	II	CC101		SRR6457842
8116	101	1/2a,3a	2009	Vegetable	II	CC101	Slow	SRR6457841
8117	9	1/2c,3c	2010	Meat	II	CC9		SRR6457815
8118	9	1/2c,3c	2010	Meat	II	CC9		SRR6457814
8119	9	1/2c,3c	2010	Meat	II	CC9		SRR6457813
8120	8	1/2a,3a	2010	Meat	II	CC8		SRR6457836
8121	8	1/2a,3a	2011	Meat	II	CC8		SRR6457831

8122	1	4b,4d,4e	2011	Meat	I	CC1	SRR6457825
8123	9	1/2c,3c	2011	Vegetable	II	CC9	SRR6457828
8124	8	1/2a,3a	2011	Environment - Floor	II	CC8	SRR6457823
8125	7	1/2a,3a	2011	Meat	II	CC7	SRR6457820
8126	321	1/2a,3a	2011	Environment - Floor	II	CC321	SRR6457864
8127	7	1/2a,3a	2011	Meat	II	CC7	SRR6457859
8128	7	1/2a,3a	2011	Meat	II	CC7	SRR6457868
8129	9	4b,4d,4e	2011	Environment - Wall	II	CC9	SRR6457857
8130	7	1/2a,3a	2012	Environment - Floor	II	CC7	SRR6457877

Only scored the B10 group, all others displayed an intermediate biofilm phenotype.

Supplementary Table 2: List of all DEGs.

This is a separate Excel spreadsheet.

Supplementary Table 3: Summary of the total 494 differentially expressed *L. monocytogenes* genes within the individual and the ST comparison at 24 and 48 h based up their clusters of orthologous groups*.

		7453#		7545#		8116#		ST101_24hr^		ST101_48hr^	
		24 hr	48 hr	24 hr	48 hr	24 hr	48 hr	Up	Down	Up	Down
CELLULAR PROCESSES AND SIGNALING											
Cell cycle control, cell division, chromosome partitioning	D	3	1	1	0	0	0	0	0	0	0
Cell wall/membrane/envelope biogenesis	M	17	2	11	0	0	0	0	0	0	0
Cell motility	N	4	0	2	0	0	0	0	0	0	0
Post-translational modification, protein turnover, and chaperon	O	2	2	2	0	0	0	0	0	0	0
Signal transduction mechanisms	T	13	0	2	0	0	0	0	0	0	0
Intracellular trafficking, secretion, and vesicular transport	U	4	0	2	1	0	0	0	0	0	0
Defense mechanisms	V	5	2	0	1	0	0	0	0	0	1
Extracellular structures	W	0	0	0	0	0	0	0	0	0	0
Nuclear structure	Y	0	0	0	0	0	0	0	0	0	0
Cytoskeleton	Z	0	0	0	0	0	0	0	0	0	0
INFORMATION STORAGE AND PROCESSING											
RNA processing and modification	A	0	0	0	0	0	0	0	0	0	0
Chromatin structure and dynamics	B	0	0	0	0	0	0	0	0	0	0
Translation, ribosomal structure and biogenesis	J	22	5	14	10	4	0	3	0	0	0
Transcription	K	16	8	4	2	2	0	0	0	0	1
Replication, recombination and repair	L	10	3	4	1	0	0	0	0	0	0
METABOLISM											
Energy production and conversion	C	11	6	1	2	0	0	0	0	0	0
Amino acid transport and metabolism	E	14	7	4	1	0	0	0	0	0	0
Nucleotide transport and metabolism	F	8	4	2	2	0	0	0	0	0	3
Carbohydrate transport and metabolism	G	34	12	7	2	0	0	0	0	0	0
Coenzyme transport and metabolism	H	8	3	1	0	0	0	0	0	0	0
Lipid transport and metabolism	I	3	6	1	0	1	0	0	0	0	0
Inorganic ion transport and metabolism	P	15	4	7	0	0	0	0	0	0	0
Secondary metabolites biosynthesis, transport, and catabolis	Q	5	2	0	0	1	0	0	0	0	0
POORLY CHARACTERIZED											
General function prediction only	R	0	0	0	0	0	0	0	0	0	0
Function unknown	S	108	19	18	1	0	0	8	0	0	1

* FDR <0.01 $\log_2$ fold change

# Differentially expressed genes in individual comparison 24 hr vs 48 hr.

^ Differentially expressed genes in ST comparison 7453 vs 8116 at 24 hrs and 7453 vs 8116 at 48 hr.

References

1 R: A language and environment for statistical computing. v. 3.4.1 (R Foundations for statistical computing., Vienna, Austria, 2017).
